# Exploring health care professionals’ experiences and knowledge of woman-centred care in a university hospital

**DOI:** 10.1371/journal.pone.0286852

**Published:** 2023-07-05

**Authors:** Lucia Floris, Benedicte Michoud-Bertinotti, Begoña Martinez de Tejada, Sara de Oliveira, Riccardo Pfister, Stéphanie Parguey, Harriet E. Thorn-Cole, Claire de Labrusse

**Affiliations:** 1 HESAV School of Health Sciences, HES-SO University of Applied Sciences and Arts Western Switzerland, Delémont, Switzerland; 2 Geneva University Hospitals and University of Geneva Faculty of Medicine, Department of Pediatrics, Gynecology and Obstetrics, Geneva, Switzerland; Lausanne University Hospital: Centre Hospitalier Universitaire Vaudois (CH), SWITZERLAND

## Abstract

Inspired by the six quality-of-care goals developed by the Institute of Medicine, woman-centred care (WCC) as model of care is used in maternity services as it gives an emphasis on the woman as an individual and not her status as a patient. Bringing stronger attention to women’s needs and values, is proven to have clear benefits for perinatal outcomes, but fails to be known or recognised by healthcare professionals’ (HCPs) and implemented. Using a mixed-methods approach, this study aimed to explore HCPs definitions of WCC and identify the degree of agreement and knowledge regarding perinatal indicators when a WCC model of care is implemented. The quantitative part was carried using a self-administered questionnaire with perinatal indicators identified from the literature. Semi-structured interviews were realized using a purposive sample of 15 HCPs and an interview grid inspired by Leap’s WCC model. The study was conducted in the maternity of a university hospital in French-speaking part of Switzerland. Out of 318 HCPs working with mothers and their newborns, 51% had already heard of WCC without being familiar with Leap’s model. The HCPs were aware of the positive perinatal care outcomes when WCC was implemented: women’s satisfaction (99.2%), health promotion (97.6%), HCP’s job satisfaction (93.2%) and positive feelings about their work (85.6%), which were strongly emphasised in the interviews. The respondents reported institutional difficulties in implementing the model such as administrative overload and lack of time. The positive outcomes of WCC on spontaneous deliveries and improved neonatal adaptation were known by most HCPs (63.4% and 59.9%, respectively). However, fewer than half of the HCPs highlighted the model’s positive effects on analgesia and episiotomies or its financial benefits. Knowledge of quality-of-care outcomes (i.e women’s satisfaction, positive impact on practice…) was prevalent among most of HCPs. Without adhering to a common definition and without a specific model for consensus, most providers have integrated some aspects of WCC into their practice. However, specific perinatal indicators remain largely unknown, which may hinder the implementation of WCC.

## Background

**Fig 1:**
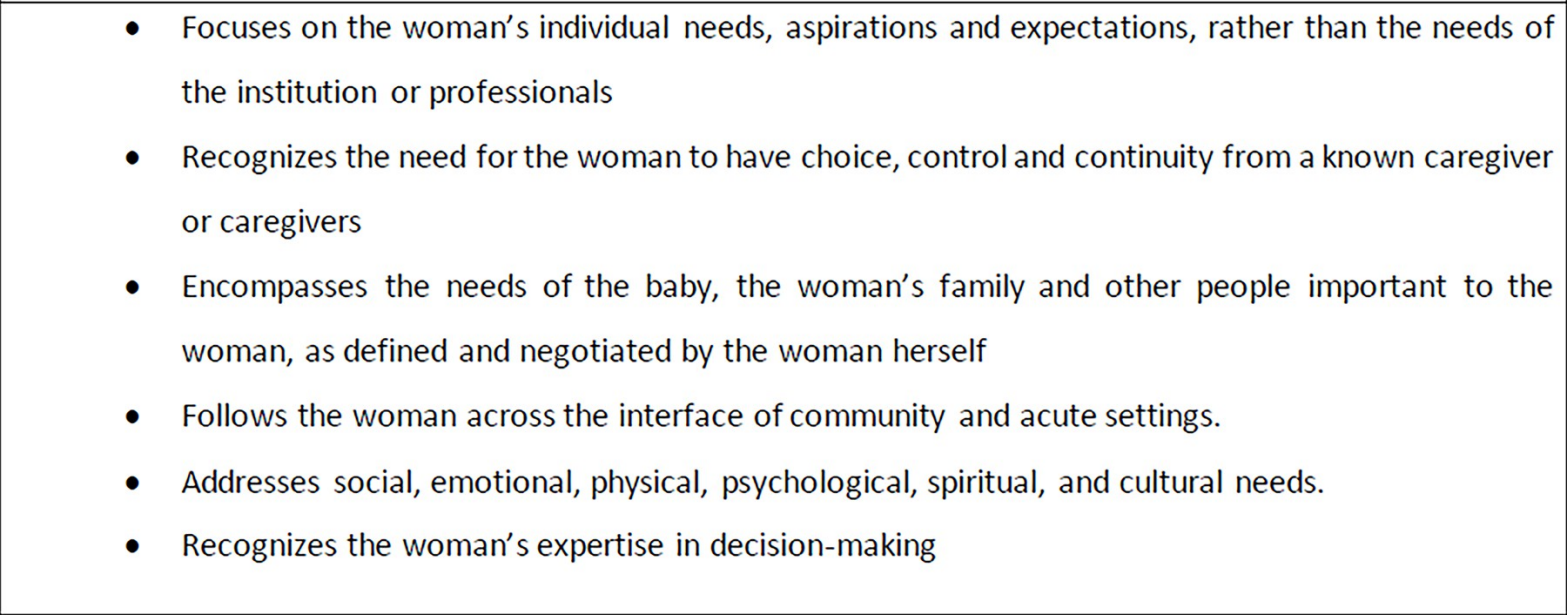
Leap’s definition of woman’s centred care [4].

Since, the development of the six aims for healthcare quality Improvement by the Institute of Medicine (IOM) [1], woman-centred care (WCC) has been used for over two decades by regulatory bodies and academics as it best fits the context of maternity care [1–3].The woman-centred care (WCC) model emerged after a series of reforms in the early 2000s to modernise the UK’s National Health Service, with a focus on individual needs and the hope that carers could work collaboratively with these women to meet their needs [4]. The WCC philosophy foundations were laid by the Royal College of Midwives (RCM) in the UK, which defined it as “an approach that puts the wishes and needs of the user first, emphasising the importance of choice, continuity of care, user involvement, clinical effectiveness, responsiveness and accessibility” [5]. Leap (2009) describes WCC as a concept that includes the need to address women’s emotional and psychological expectations on a continuum of care [6,7]. According to Leap’s (2009) definition, WCC adopts a holistic approach whilst enabling the woman to take control of her health (Fig 1).

The implementation of WCC has benefited perinatal care and resulted in improvements to several perinatal quality indicators, including fewer unnecessary medical interventions such as epidural and episiotomies during childbirth [8,9] with no differences in neonatal outcomes, such as Apgar scores and neonatal intensive care unit admissions.

The benefits of WCC in maternity departments are multiple. The Cochrane Review, involving 15 randomised clinical trials (N = 17,674), described that women who received WCC reported less epidural analgesia at delivery (RR = 0. 85, 95% CI 0.78; 0.92) and fewer episiotomies (RR = 0.84, 95% CI 0.77; 0.92), comparing with women receiving other models of care (i.e in traditional labour wards), among women who received midwife-led care. In the same study, the results showed more spontaneous deliveries (RR = 1.05, 95% CI 1.03; 1.07), N = 16,687, compared to the usual care group. There were no differences between the different types of follow-ups in neonatal outcomes, such as Apgar score or the number of admissions to an intensive neonatal care unit [8].

While favourable elements of birth outcomes have been recognised, an increase in women’s satisfaction has also been reported. Several studies in Anglo-Saxon and Scandinavian countries have shown high satisfaction rates among women who have received WCC [8,10]. In a randomised clinical trial in Australia, 71.2% of women in the continuing care group in hospitals (697/979), compared with 62.6% of women in the usual care group (516/824), reported a positive childbirth experience (adjusted OR = 1.50, 95% CI 1.22; 1.84) [11]. For women, midwife-coordinated care enhanced their own sense of self-worth [12]. Floris et al. (2017), in a study carried out in a University Hospitals of Geneva (HUG) with 350 women, showed a higher satisfaction rate in the group whose care was coordinated and monitored by a group of midwives providing continuity pf care compared to the usual care group, whose care was shared between doctors and midwives. The higher satisfaction rate was reported for all episodes of care (prenatal, delivery and postpartum). In the same study, a secondary analysis using Swiss Diagnostic Related Group classification found no additional costs between the continuity of care model and the traditional care model [13].

Studies have illustrated that midwives working with a WCC model have reported higher job satisfaction, better cohesion, more satisfying interactions with women and less burnout than midwives working in traditional organisations of care [14–16]. Most studies have reported the important role that midwives play in WCC, particularly in encouraging childbirths without complications, or of independent midwives facilitating childbirth at birth centres or physiological units within hospitals [17,18]. However, most women lack access to personalised care from midwives during the perinatal period. To fill this gap, Keedle [9] and Shaw [19] advised to further implement WCC in hospitals. Nevertheless, this should be done with the best care possible as some reports of disrespect during maternity care from women and their partners highlight a gap between the theoretical definitions of WCC and caregivers’ practices [20–22].

Another aspect seldom investigated is the experience of HCPs when the care is shared across a multidisciplinary team. Indeed, most studies explore midwives’ perception and not that of other HCPs like obstetricians or paediatricians. Their experience of practicing WCC, their knowledge of the model and its impact on the organisation, is yet to be explored [23].

Maillefer et al. (2015) underlined the necessity of building efficient interprofessional collaboration between different HCPs for the implementation of WCC. In their study, some HCPs have shown difficulty respecting the physiological framework specific to midwifery or have struggled to collaborate with others in pathological situations, which points the importance of the continuity of care [24].

WCC is a relatively new concept. Brady’s et al. (2019) study presented a large panel that explored how significant differences and misunderstandings in HCPs’ interpretations of WCC can arise, as each professional has a different way of defining and dispensing it [25]. Finally, most studies often put forward the absence of WCC but without questioning how and to what extent the approach can be implemented within labour care in a hospital setting [18,26,27].

The purpose of this study was to explore the global perception of WCC from HCPs of different specialities and their views on facilitators and barriers to the implementation of this model to their practice. Woman-centred care (WCC) in this article has been defined as care that is focused on the woman, her newborn infant and her family/relatives; this includes her partner, male or female, her family and her relatives (self-designated). The data collected were divided into three parts according to their specific theme. Due to the amount of data, the authors will be presented in other communications. The quantitative and qualitative data (written comments and interviews) were analysed according to the following elements:

1. Knowledge and degree of agreement of HCPs with perinatal outcomes of WCC as reported in the scientific literature (sections A, B, C and E of the questionnaire)
2. Barriers and facilitators (components F and G of the questionnaire) with the qualitative part, written comments and interviews
3. Centred-care practice (component D of the questionnaire) and attitude in practice with the qualitative part, written comments and interviews

This article will only report on part 1.

The research questions were the following:

1. What is the HCPs’ awareness of WCC?
2. How do HCPs define WCC?
3. How do HCPs compare their own definition to Leap’s definition of WCC?
4. To the HCPs’ perception, how do WCC influence perinatal outcomes?
5. To what extent do HCPs agree with WCC impact on perinatal outcomes?

## Methods

### Design of study

A sequential mixed method approach of an explanatory nature was chosen [28,29] to determine the points of view of the HCPs implicated in perinatal care upon implementation of WCC. First, a cross-sectional study with a self-administered, computerised and anonymised questionnaire was used to collect a maximum of information from a large sample. It was completed with two open questions based on the Leap model of WCC [6], and then, by semi-structured interviews in a sample of HCPs to elaborate on some responses of the questionnaire and deepen understanding.

### Place of study and participants

The study was conducted within the Department of Women, Children and Adolescents, and in collaboration of the Anaesthesiology Division, of Geneva’s University Hospitals in Switzerland. More than 4,000 babies are born in the hospital annually from healthy women with straightforward pregnancies as well as women living with a pathology or with a complex pregnancy. The department also includes a Neonatal and Paediatric Intensive Care Unit (NICU), enabling the provision of care to all, healthy and sick neonates. All HCPs who care for women and neonates in the Department’s maternity ward and NICU were eligible to participate, thus the sample included obstetricians, anaesthetists, paediatricians, nurses, and midwives. The hospital mostly employs HCPs who trained in Swiss or in European Union schools or universities, resulting in a variety of practices and experiences within the institution in relation to WCC approach.

### The quantitative component

#### Data collection

Leaflets containing information on the purpose and conduct of the study were distributed to the HCPs in their departments, and information sessions were held in the various units of the Department of Women, Children and Adolescents, and of the Anaesthesiology Division. The questionnaire was accessible via a hypertext link inserted in an invitation letter sent by e-mail to the participants’ professional e-mail addresses, which were obtained from the chief of staff medical doctor, the midwifery and the nursing team managers. Two further e-mail reminders were sent out after the questionnaire was disseminated. HCPs who did not wish to participate in the study could select ‘do not wish to participate in the study’, and the HCPs to whom the study did not apply because they did not work in the perinatal area could select ‘do not work, or no longer work with mothers and newborns’.

The electronic link to the questionnaire was valid for the duration of the study from March 2019 until July 2019. According to a meta-analysis of 48 studies about techniques to improve survey response rates by health care professionals, without incentives the average rate is 48%, which could rise to 60% with financial incentives [30]. To ensure adequate participation, this study was carefully planned (i.e. including reminders, available paper, regular requests to health care professionals in the services to complete the questionnaires and presentations to supervisors to explain the project) to achieve an adequate response rate of at least 60% [31].

#### Development of the questionnaire

In the absence of a validated tool on the subject, a questionnaire was developed on the basis of data from the literature [1,6,8,24,32–34]. The questionnaire included 7 sections: (A) socio-demographics characteristics, (B) knowledge about WCC, (C) comparison between the participants definition of WCC and Leap’s definition, (D) agreement with components of WCC as described by Leap (6), (E) agreement with impacts of WCC as reported in the literature, (F) barriers to the implementation of WCC, (G) facilitators to the implementation of WCC (Annexe 1). This article will present only sections A, B, C and E of the questionnaire. Knowledge (B) and degree of agreement (E) were assessed with thirteen items, ten of which were expressed as positive statements, and three of which were negative. Responses to these statements were rated on a Likert-type scale with five categories: strongly disagree/disagree/neither agree nor disagree/agree/strongly agree. These categories were then grouped into two dimensions for analysis: positive (agree/strongly agree) and others (neither agree nor disagree/disagree/strongly disagree). The option of “neither agree nor disagree” in the “others” dimension signified not knowing the estimated indicator. The percentage of positive responses indicated a knowledge of the favourable impact of WCC on perinatal indicators (or the inverse for negatively worded items). This statistical dichotomy was supported for the measure used for safety culture of care [35].

HCPs who considered not being concerned by the item were given the option to respond, ‘Does not apply to my activity’. (C) comparison between the participants definition of WCC and Leap’s definition was assessed using thematic analysis.

The content of the questions was examined by a committee comprised of four midwives and four medical doctors (two obstetricians, 1 paediatrician, 1 anaesthetist) and by the Clinical Research Centre, University of Geneva and Geneva University Hospitals. The pilot questionnaire was pre-tested with a representative sample of the target population [36] using cognitive interviews with focused questions that were designed to facilitate understanding of the questionnaire. The number of pre-tests was determined based on elements of the questionnaire identified as problematic [37]. The first phase of the pre-test was conducted with 10 HCPs with a similar profile, and then the pilot questionnaire was modified, taking into account the participants’ observations. The final questionnaire was then retested by 10 other HCPs, and also sought socio-professional demographics including age, occupation, position held, number of years working in the institution, activity rate, continuing professional development, current ward placement. Prior to the dissemination of the survey, the hyperlink was tested to ensure its functionality.

Lime Survey software was used for computer support (LimeSurvey^©^, v3.27)

#### Statistical analysis

The global participation rate was calculated according to the following recommendations [38]:

$$Participation\;rate=\frac{Number\;of\;questionnaires\;returned-incomplete\;questionnaires}{Number\;of\;questionnaires\;distributed-ineligible\;questionnaires}$$

The number of questionnaires distributed was the number of e-mails address used. The ineligible HCPs were defined by automatic failed delivery emails or emails written by an HCP indicating he/she no longer worked in the area of perinatal care. Finally, the number of HCPs who had taken a leave of absence by unit were communicated to the research group by the unit lead.

Incomplete questionnaires were defined by the number of participants who only partially completed the questionnaires. For each section and item, the participation rate will be reported. Participants who selected ‘does not apply to my practice’ will be reported by item and by profession.

Descriptive analyses (including mean, standard deviation, number, frequency, median, and quartiles) were used for the analysis of the dependent and independent variables. All missing data were reported. Chi-squared tests were used to test the relationships between categorical variables. A p-value < 0.05 was considered statistically significant. The data were transferred to SPSS IBM 25.0 software, and the graphs created with the statistical software Stata: Release 12.

### The qualitative component

#### Data collection and analysis of open-ended questions

Along with the quantitative component of the questionnaire, two optional open-ended questions were included to explore HCPs’ definitions of WCC, and how participants compare their own definition to that of Leap’s (2009) and the items presented in Fig 1.

1. What would be your definition of WCC?
2. What do you think of Leap’s definition in relation to what you mentioned earlier?

The textual data was thematically coded with similarities, differences and complementing aspects between the theoretical definition and that of the participants identified.

#### Data collection and analysis of one-to-one interviews

At the end of the questionnaire, HCPs were invited to insert their email address or contact the research team directly if they wished to participate in the next stage of the study, a one-to-one in-depth interview. A purposive sampling of 15 was chosen to ensure all professions are proportionally represented: seven midwives, two midwives or nurse team managers or advanced practitioners, two junior obstetrician gynaecologist, two senior obstetrician gynaecologist (senior doctors), one paediatrician, one anaesthetist.

The interview guide was formulated based on a specific literature review [6,10,32,33] and on the preliminary results of the quantitative data analysis. Open-ended questions were expected to be used to deepen understanding of participants’ perception and daily experience of WCC. The topic guide and process were pilot-tested by two HCPs who met the eligibility criteria and was readjusted. After three interviews with participants, the topic guide was slightly revised to make it more comprehensive and to ease participants’ answers.

The time and location of the interviews were organised according to the participants’ preferences, and written consent was obtained from all participants prior to commencing the interview. All interviews were audio transcribed verbatim, anonymised, and reread with the audio by CDL, BMB and LF to ensure accuracy and compare the concordance.

The data, originating from semi-structured interviews, was thematically analysed with MAXQDA data management software©. Thematic analysis was used to analyse semi-structured interviews as it permits to transparently link the analysis process and draw out the participants’ views, opinions, knowledge, and experiences [39,40]. Then, key themes were identified, and a coding framework was developed by CDL and BMB. This was presented to the research team to ensure the rigour of the analysis process. Questions and divergences were discussed amongst all the authors.

#### In the mixed analysis

Once the data from each section was analysed, the quantitative descriptive results were combined with the analytical results from the HCPs’ perspectives for comparison, contrast or association. This tandem processing of the data generated results with greater overall strength than those obtained from two separate studies [29].

#### Ethical considerations

This research was presented to the Cantonal committee of ethics of research who approved the study (2018–00826). Approval for e-mail identification of the participants was obtained from the direction of the hospital. Before the start of the study, each HCP received a flyer and e-mail with information on the aim and involvement of the study and the contact details of the researchers to ask questions. Within the email, before the survey started two buttons “I participate in the study” and “I do not wish the participate in the study” were proposed, with the first consisted of the participant to consent to participate. All questionnaires were anonymised. An invitation to participate to an interview was sent to the participants who completed the questionnaire. Consent form was provided, explained and signed before doing the interviews.

## Results

**Fig 2:**
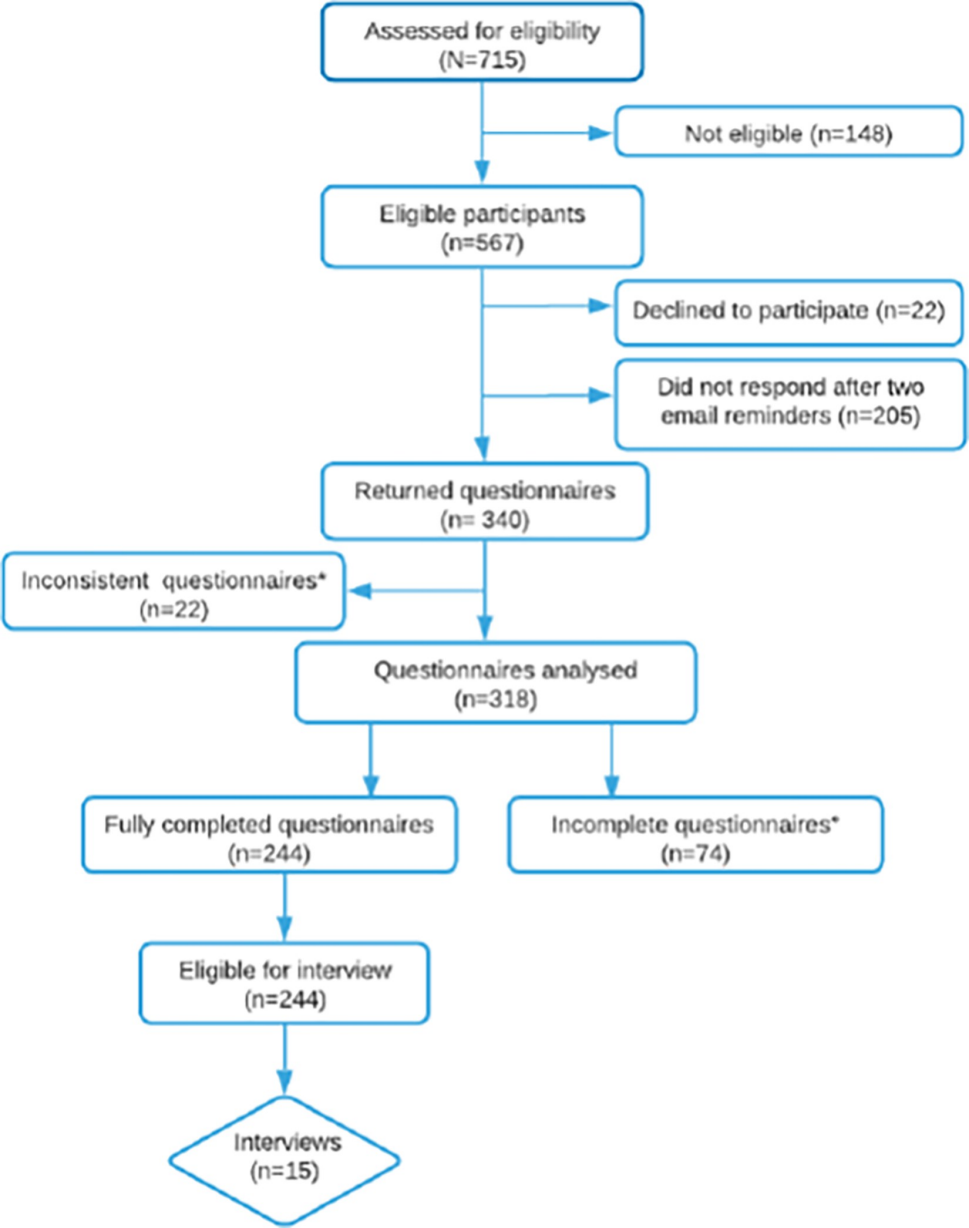
Participant diagram. * Thirteen open/closed parts of the questionnaire, and nine questionnaires where the first page was not fully completed. ° Incomplete questionnaire.

Among the 715 HCPs’, 148 were defined ineligible (91 were no longer working in the area of perinatal care at the time of the study, 57 HCPs were absent from the institution due to illness, accident, maternity or other personal reasons). Fifteen HCPs were interviewed (Fig 2).

The eligible HCPs sample included 407 (71.8%) midwives and nurses, and 160 (28.2%) medical doctors, half of whom were gynaecologists-obstetricians. The participation rate of eligible HCP was 56.1% (n = 318) and their distribution by profession is detailed in Table 1.

**Table 1:** Participation rate of eligible participants by profession (N = 567).

Profession	n/N (%)
Midwives	137/206 (66.5)
Nurses	119/201 (59.2)
Gynaecologist-obstetrician	31/82 (37.8)
Paediatricians	16/42 (38.1)
Anaesthetists	15/36 (41.1)

The participation rate of midwives and nurses was higher than that of other medical professions, 256/407 (62.9%) for the former and a rate of 62/160 (38.8%) for the latter (χ2 = 19.68, dl1, p = 0.002) (Table 1). The socio-professional profiles of the participants who fully completed the questionnaire and those who completed only part of it are presented in S1 Table.

### Socio-demographic, professional profile of participants and knowledge

The average age of the participants was 40.6 years (SD = 9.5), with a median age (IQR) of 40.0 years (Q1–Q3); 115 (36.1%) participants were under 36 years of age, 101 (31.7%) were between 36 and 45 years of age and 103 (32.3%) were over 45 years of age; 296 (92.8%) participants were female. The socio-demographic characteristics of the participants are presented in Table 2.

**Table 2:** Socio-professional characteristics of participants and awareness of WCC (N = 318).

	All participants	Knowledge of WCC		
		Yes	No	
	n (%)	n (%)	n (%)	P
Professions				
Midwifery	137 (43.1)	79 (57.7)	58 (42.3)	0.05
Nursing	121 (38.1)	61 (50.4)	60 (49.6)	
Gynaecologist-obstetrician	31 (9.7)	13 (41.9)	18 (58.1)	
Paediatrician	16 (5.0)	8 (50.0)	8 (50.0)	
Anaesthetists	13 (4.1)	2 (15.4)	11 (84.6)	
Country of graduation				
Switzerland	154 (48.4)	81 (52.6)	73 (47.4)	0.65
Other	164 (51.6)	82 (50.0)	82 (50.0)	
Position held				
Midwife	116 (36.5)	65 (56.0)	51 (44.0)	0.005
Nurse	112 (35.2)	52 (46.4)	60 (53.6)	
Midwife or nurse team managers and Advanced Practitioners	30 (9.4)	23 (76.7)	7 (23.3)	
Junior doctors	26 (8.2)	8 (30.8)	18 (69.2)	
Senior doctors	34 (10.7)	15 (44.1)	19 (55.9)	
Continuing education				
Yes	118 (37.1)	59 (50.0)	59 (50.0)	0.82
No	200 (62.9)	104 (52.0)	96 (48.0)	
Professional Experience (years)				
< 9	112 (35.3)	66 (58.9)	46 (41.1)	0.82
9–19	107 (33.8)	47 (43.9)	60 (56.1)	
> 19	98 (30.9)	49 (50.0)	98 (50.0)	
Activity rate (%)				
90–100	154 (48.4)	81 (52.6)	73 (47.4)	0.42
< 90	164 (51.6)	82 (50.0)	82 (50.0)	
Gender				
Women Men	295 (92.8) 23 (7.2)	151 (51.2) 12 (52.2)	144 (48.8) 11(47.8)	0.50

### Knowledge of WCC model

More than half of the HCPs (163; 51.3%) had already heard of WCC. However, the results showed a disparity in awareness of WCC among HCPs. More than half of the midwives (79; 57.7%) had heard of WCC, whereas only 41.9% of gynaecologist-obstetricians had, but this difference was not statistically significant. In paediatrics and neonatology, half of the nurse practitioners and paediatricians had heard of WCC. Anaesthesiologists, on the other hand, had little awareness (15.4%) of this approach to care (Table 2).

By occupation, midwifery and nursing team managers and advanced practitioners were more often aware of WCC compared to those in the other positions (χ2 = 14.92, dl 4, p = 0.005). The other main indicators, such as length of practice and rate of activity or continuity in education, did not show statistically significant differences. A large proportion of participants answered the two open-ended questions: 91,8% of participants gave their definition of WCC, and 88,1% compared their definition with Leap’s.

Some Participants used these opportunities to either confirm their lack of awareness:

*‘I don’t know’ (Comment 63 of the open-ended questions in the questionnaire)*

*‘?’ (Comment 124)*

or to propose their own definitions, in short or extended ways, or only in reference to their daily practice:

*‘global accompaniment’ (Comment 104)*

*‘totality of care provided for optimal follow-up of the woman and the newborn, also including the spouse and the extended family, at the physical, emotional or even spiritual level. A more personalised follow-up allowing a real relationship of trust, by listening to the needs and desires of the patient. And a process with a patient who is involved and able to participate in the decision-making process.’ (Comment 92)*

*‘What I do on a daily basis’ (Comment 172)*

When writing their own definition, the majority of participants referred to one or more elements of the WCC definition from Leap (2009), but none of them provided the full definition. Individualisation of care and comprehensiveness were the most frequently mentioned elements:

*‘[…] care adapted and personalised to families according to their values, lifestyle and activities’ (Comment 74)*

*‘the bio-psychosocial and cultural globality of the woman, her needs, her baby and her entourage at the heart of care’ (Comment 145)*

In contrast, women’s need to have control and their expertise in informed decision-making were almost absent from those definitions. And when these points were mentioned, HCPs saw women as participants in decision-making rather than experts. HCPs positioned themselves as those who ’give’ the responsibility or opportunity to women to participate in their care, thus retaining control.

*‘It is care that involves all the actors in the family, to make them responsible for their health and situation by making them actively participate in all stages of pregnancy, childbirth and the postpartum period.’ (Comment 255)*

*‘…parents are actively involved in making decisions about their child’s care in close collaboration with caregivers.’ (Comment 422)*

This trend was found in the interviews as well, where decision-making was discussed more extensively and offered to understand that although HCPs are open to shared-decision making practical reality might be very different.

*"But I would say that the whole team still seems much more open to the fact that women should have more choice" [Interview 3]*.

*"That way, I would say, we don’t recognise women’s skills in decision making. Most of the time. I don’t really feel that much. It’s not so much in our culture, um… We know in her place… We know in her place…" [Interview 12]*

*"But I think that sometimes we are right when we override the patients’ wishes. » [Interview 4]*

Participants also highlighted important elements to take into account, which were not stipulated in the definition from Leap (2009), like women’s own resources, history, previous experience, context and environment.

*‘Care centred on the needs of the person with his or her beliefs and individuality. Respect for the customs of fundamental rights. Include possible family resources in the care process.’ (Comment 372)*

*‘care that takes into account the totality of the woman, the newborn and the family, taking into account the socio-economic and/or family environment.’ (Comment 285)*

### Effects of WCC on perinatal outcomes

Of the 270 HCPs who participated in the questionnaire on positive perinatal outcomes, 144 (53.3%) completed all 13 items. Amongst these participants, a high proportion were midwives (101; 70.1%) and Obstetrician-gynaecologist (18.8%). Other professionals accounted for 11.1% of the participants, including nine nurses (6.2%), four anaesthetists (2.7%) and three paediatricians (2.1%).

**Fig 3:**
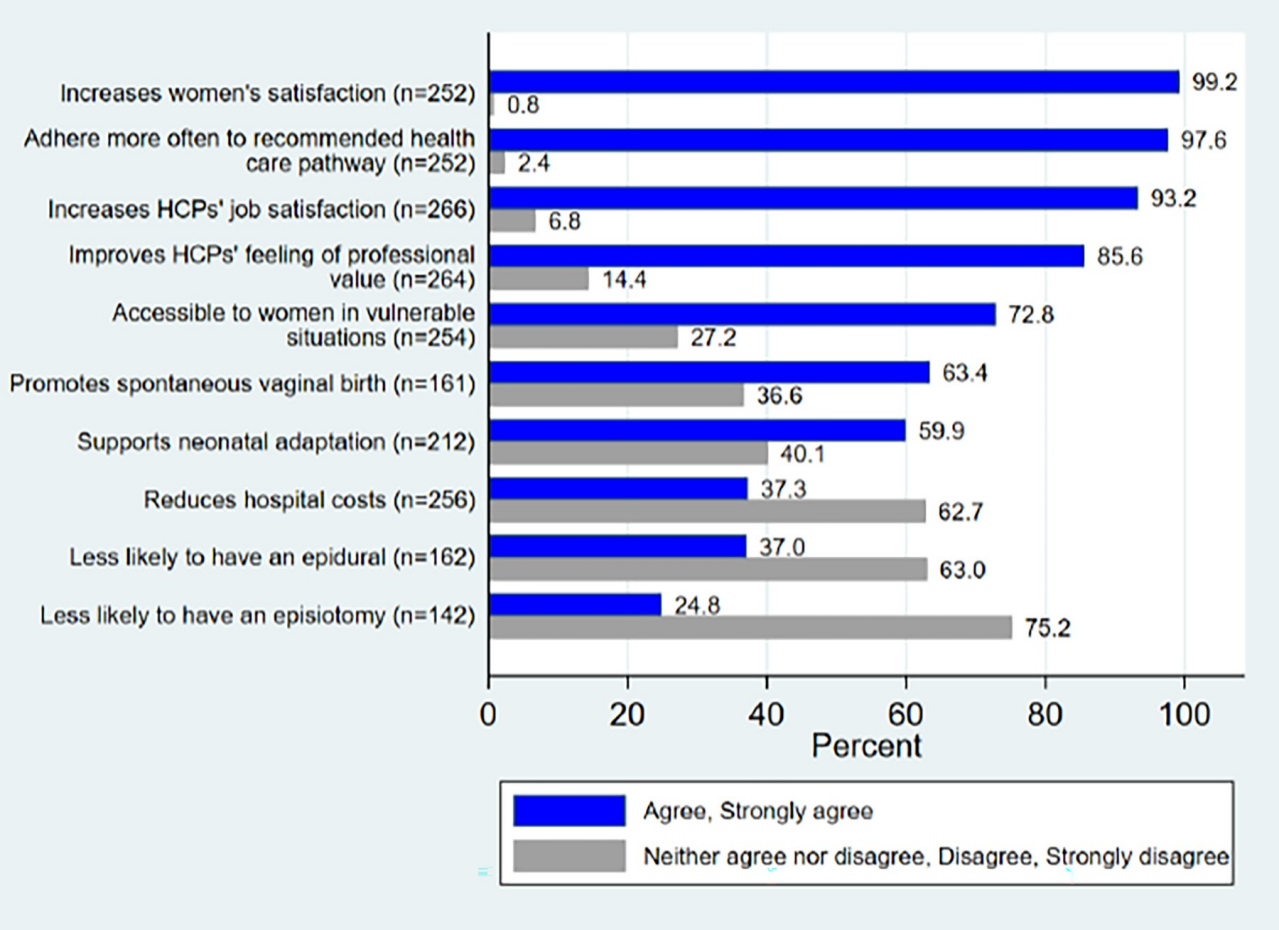
Knowledge and degree of agreement of HCPs with perinatal outcomes of WCC, positively statements.

More than 97% of the HCPs acknowledged that WCC could contribute to women’s satisfaction and encourage their adherence to the recommendations for their own and their child’s health. A total of 72.8% of HCPs specified that WCC should be accessible to women in vulnerable situations. With respect to childbirth, more than half of the HCPs believed that this practice helps support spontaneous vaginal deliveries (63.4%) and improves neonatal adaptation (59.9%), and does not increase the risk of Neonatal Intensive Care Unit admission (73.6%). However, the potential impact in reducing hospital costs was agreed upon by only 37.3% of the HCPs (Fig 3).

Only 37% of all HCPs believed that WCC reduces the rate of epidural anaesthesia and episiotomies. With respect to the workplace and working relationships, 85.6% of HCPs reported that the practice of WCC could improve their sense of professional value and increased job satisfaction, and 70.1% believe that the practice would not increase burnout at work.

**Fig 4:**
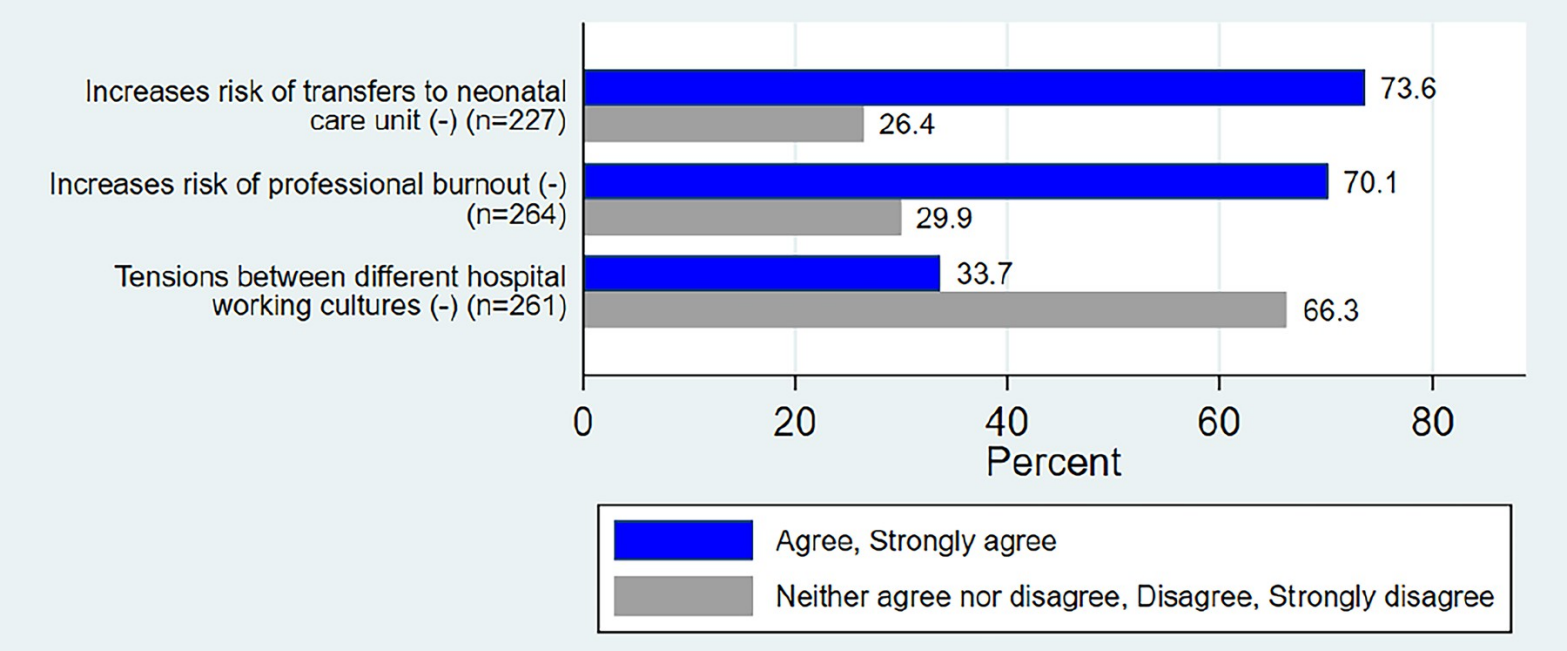
Knowledge and degree of agreement of HCPs with perinatal outcomes of WCC, negatively turned statements.

However, 66.3% of HCPs pointed out the possible tensions generated by the practice of this model of care (Fig 4).

The descriptive analysis of the responses by profession of knowledge and degree of agreement of the effects of WCC on perinatal outcomes is shown in S2 Table.

Qualitative results strengthened the results obtained from the positive statements of the quantitative component of the survey, especially in regards to women’s global experience. HCPs believe that WCC improve their relationships and collaboration with parents and other HCPs.

*"When we integrate parents into care and make them autonomous and then make them actors, it’s… First, the relationship we have with them is much better, there is much less conflict, there are much fewer complaints […] So if we integrate them well from the start, we can clearly see the difference. "[Interview 9]*

As in the quantitative part, WCC is recognised to improve health, suggesting a higher quality of care.

*"But we also know that with parents who are there 24 hours a day, there is less pathology, weight gain is better, and children go home earlier." [Interview 7]*

Job satisfaction was another important theme extracted from the qualitative part, echoing the quantitative survey results. Interviewees spontaneously referred to extremely positive experiences when they are able to provide WCC.

*"When you have the time, and then you’re really in that… you’re really in active listening, where you really let people express themselves fully and then accompany them, you have the impression… that you’ve had an excellent day. » [Interview 2]*

*"Ah, it’s super… it’s super gratifying. » [Interview 8]*

For those participants that were already concerned by internal projects which were inline with WCC in their unit, changes observed in their practice were strongly positive as well.

*"I feel vaguely like at home, with this knowledge of people, where I have the impression that they’re doing their own thing and then I’m next door, like at home in fact. So for me it’s changed everything. "[Interview 3]*

*"Things have really changed and when you try to make a decision with the patient, in the end the patient’s feedback is more satisfying. It’s a closer relationship and even if we have a bad result, the patient’s acceptance is better than if we had made the whole decision. So in a way it is a shared thing. "[Interview 14].*

However, not everything seemed totally straight-forward as the increase in time spent, energy and responsibilities was pointed out as a challenge, not always compatible with the institution’s management.

*"I try to do it, that’s why sometimes it takes me a lot of time to talk to women but I try to do it and… But it takes time, it’s true… it takes a lot of time because, on the one hand, you have to create this atmosphere of trust…” [Interview 14]*

*"It means… it means jumping into the deep end, it means taking responsibility. It means being a little less at ease, taking responsibility for being less at ease." [Interview 12]*

Nevertheless, some of the participants chose to favour this type of care model, sometimes to the detriment of their rest time.

*"Today, I really try to do it to a maximum but I’m actually taking over the administrative part, so I keep it to myself in the evenings. So it actually takes on my own state of fatigue, in fact. " [Interview 6]*

Finally, although tensions due to WCC practice were not explicitly expressed in the interviews, participants did address differences in working culture. Education, institutional expectations or work organisation are some of the elements that seem to lead to such differences.

*“They [the doctors] are totally formatted precisely on technique, profitability, finding the pathology. In fact, they are not trained to provide support. They often, very often, have a lot of difficulties." [Interview 6]*

*“When there is something wrong, they [the doctors] will be a little sensitive, yes. But not in the same way as us. But we are there for twelve hours, or eight hours with this family, with this baby. They are there on an ad hoc basis.” [Interview 8]*

#### Not applicable to my activity

**Fig 5:**
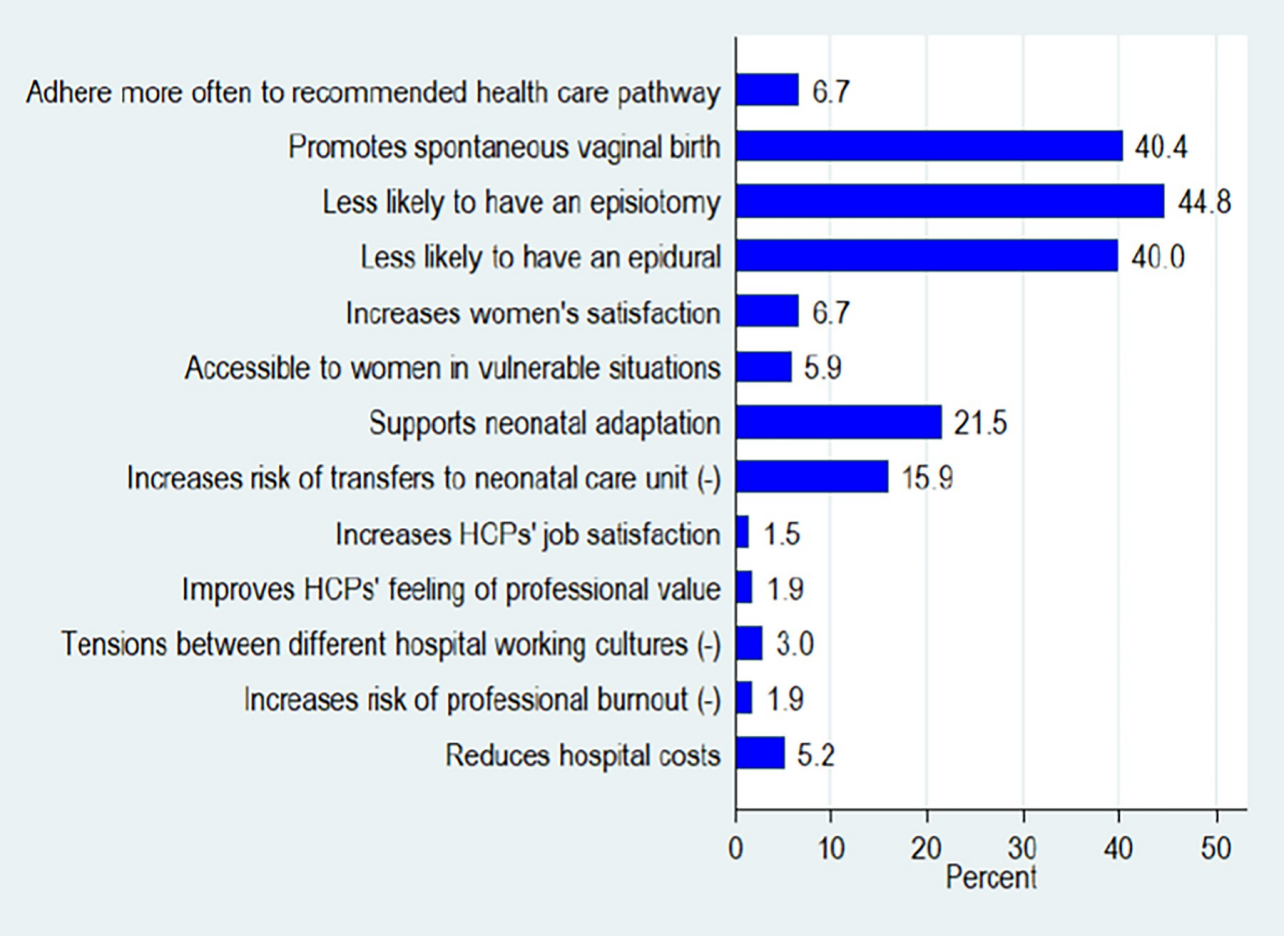
Percentage of all respondents for the option, ‘not applicable to my activity’ (N = 270).

Of the HCPs (n = 270) who responded to the questions regarding perinatal outcomes of WCC and their perceptions, between 1.5% and 44.8% (Fig 5) chose the option *not applicable to my activity*. The items most affected by this response option were those related to childbirth (spontaneous vaginal, epidural and episiotomy) and were reported by nurses and paediatricians whose major activity is not in the labour ward. Surprisingly, 21.5% of respondents felt like the item “WCC supports neonatal adaptation” and 15.9% felt that “WCC increases the risk of transfer to the neonatal care unit” was not applicable to their practice.

The results for each item are detailed in Fig 5 (Descriptive analysis of all respondents by profession for the option, ‘not applicable to my activity’ are in S3 Table).

## Discussion

The key findings of this research are that almost 50% of all HCPs have never heard of WCC before. But amongst those HCPs, midwives had a higher awareness of what it encompasses. Written comments confirmed the lack of general awareness on the definition of WCC, even with some HCPs trying to find synonyms. Participants reported in Leap’s definition the lack of acknowledgement of women’s resources. In relation to WCC ‘s impact, between 97% and 85,6% agreed that WCC improves midwives’ and women’ satisfaction, this was comforted by HCPs ‘quotes. Two third of the participants expressed that this model could lead to tensions between hospital working cultures. Mostly HCPs did not know that rates of epidural, episiotomies and costs could be reduced by WCC. Rightfully 77% of HCPs approved the fact that WCC reduces the risk of transfers to neonatal care unit and 59,5% agreed that WCC would support neonatal adaptation.

### Awareness of the definition of WCC

In this study, only half of the HCPs had heard about WCC, with a majority being midwives. The analysis of HCPs’ definition showed the difficulty to put words on a definition of what is providing care that is centred on the woman and her newborn, which includes all the elements identified by Leap [6]. The general ideas and terminology reported by the participants are diverse and demonstrate a rich and extensive lexicon, reaching the main point of Leap’s definition [6]. The open-ended questions of the first part of the questionnaire demonstrated that most HCPs have identified WCC as an overall concept that include everything women needs to have. However, the in-depth analysis of the interviews brought to our attention that the application of WCC varies amongst each HCPs’. Individual subjectivity on how WCC should be delivered induces differences from one another [18]) and therefore not a subject of agreement as underlined in the review study and the integrative literature review of Brady [25,41].

The sixth element of Leap as “recognizes the woman’s expertise in decision-making” was the most absent from the HCP’ definitions, but when it was provided woman’s expertise in decision making had to be in accordance with the HCP. In the present study, several HCPs describe WCC as “a process [in] which the patient is involved and able to participate in the decision-making” and includes the personalisation or individualisation of care or education according to the patient’s needs. This finding was also reported in the Hunter study [42], which underlines the need to provide an environment favourable to normality that facilitates women’s access to WCC.

The understandings of WCC varied amongst HCPs. Midwife or nurse team managers and advanced practitioners had a higher rate of positive answers than doctors (juniors and seniors) or nurses (76.7% versus 38,3% or 46.4%).

An explanation to this lack of understanding from the medical staff was provided Roudebush (2016) and Baas (2012) highlighted that doctors were more familiar with the concept of patient-centred care [43] and less with the concept of care oriented specifically to women during the perinatal period [27,44]. Moreover, Paediatricians and nurses are more familiar with care centred on the family compared to WCC [27,44]. Another explanation for our findings might be that despite the significant development of the scientific literature over the last 10 years emphasizing the specific characteristics for the care of women [2,33], the implementation of a WCC approach in maternity services is still challenging with the difficulty in promoting WCC and the lack of involvement of users in health politics ([6,17,45,46].

Interestingly, when analysing Leap’s definition, HCPs added the importance to take into account women’s resources and background. This element not only is missing in Leap’s definition but as well in other models of care. In our study, HCPs reported the necessity of considering individual resources, such as the woman’s personal and medical history, the sociological and economic context, thus agreeing with the IOM’s recommendations [1].

Midwives’ testimonies have reported, when talking about interprofessional practice, that the WCC perspective is less known in the context of biomedical approach which is based on risk selection [17,47]. Indeed, there is a gap in the literature about the practice of WCC by medical staff such as obstetricians and paediatricians. For example, the NICE Antenatal Care Guidelines (2021) makes reference to how to communicate, provide support, respond to the women’ needs, which are components of the WCC, but without mentioning a specific model to base the provision of care [48]. WCC practice is often associated with the midwifery model of care [18], as the scope of practice covers a holistic integrated care in physiological pregnancy and childbirth. Obstetricians are also associated in this model of WCC according to the literature, but mostly when their expertise is needed which is when complications arise [42].

Some HCPs defined WCC as ‘global accompaniment” and “care adapted and personalised”, identified as a means to build a real relationship of trust. This was also reported by several clinicians in Hunter‘s qualitative study [42]. This relationship of trust has been acknowledged to encourage physiological support at birth [49]. In this research, several HCPs pointed out the need to include in the care provided the physical, emotional or even spiritual needs of the relatives (spouse and large family). These orientations have been also underlined in Saftner’s study [49].

### Knowledge of main indicators of WCC

Despite the abundance of literature on the subject, the effects of WCC on specific issues [8,50,51], such as the increased incidence of spontaneous childbirth, reduced use of epidurals or episiotomies and the reassuring consequences of WCC on newborns were unfamiliar to the HCPs, along with the financial aspects of WCC.

HCPs’ knowledge of the effects of care on perinatal outcomes is a major element in considering women (and their relatives) as stakeholders in their own care. The results of this study show that the majority of HCPs (between 97% and 85,6%) believed that WCC has an impact on women’, HCPs’ satisfaction and professional value, but 66,3% felt that it could lead to professional tensions between HCP. HCPs did not perceive that in a context of WCC, women were more likely to have less interventions such as episiotomies and epidurals (63% and 75,2% respectively) and that costs would be reduced (62,7%). The potential positive effects of WCC on women and their families, as demonstrated in the responses to the items on adherence to public health recommendations and women’s satisfaction with health care, have been recognised by the HCPs. They recognized as well the positive impact of WCC with a possible protective effect to burnout. Finally, HCPs agreed that WCC improved likelihood of spontaneous vaginal delivery and neonatal adaptation. In their statements, participants highlighted the extra time needed to practice WCC and therefore the negative collateral effects such as stress, overtime, and fatigue.

Not having a full picture of all the positive effects of WCC by HCPs could be explained as some midwives and nurses may have not received training of interpretation of scientific findings and evidence-based medicine [52,53]. If advanced practitioners midwives, nurses or nurse team managers have a higher level of knowledge of WCC, this was not the case for all midwives. Advanced practitioners midwives, nurses or nurse team managers have been trained at master’s level, which includes training in scientific literature [54]. Another element that should be considered in relation to the accessibility of scientific literature is that of language barriers as most of literature about WCC is in english [54]. The lack of knowledge on some effects of WCC could have an impact on the assessment of the main positive outcomes of WCC.

In a study undertaken in three maternity units in Ireland with participants recruited from a variety of professional grades, settings and models of care, Healy (2017) argues that midwives and obstetricians often cannot promote normal birth because they are influenced by a pathological conception of childbirth. The findings of this study reported a lack of awareness about WCC reducing some interventions, this might explain that birth has become more medicalised and risk-oriented [55].

In our study, two-thirds of HCPs reported that working cultures based (or not) on risks could cause stress and tension, which limits the possibility of implementing WCC. The need for the same philosophy and values is particularly important in healthcare services. Changes in approach, even beneficial ones, can destabilise HCPs if the framework is not clearly defined by the institution. Any changes to the institution’s values and mission should be made by consensus. As reported in the results, Hunter et al. (2017) illustrate the tension created by the co-existence of two different approaches with a “‘with-woman’ non-interventionist approach along with more medical ‘with institution’ approach” in the same institution [26]. In addition, Hunter et al. (2017) maintained the need for a shared vision: “Professionals need to develop their own knowledge, along with developing a shared ethos of pregnancy and childbirth. The lack of a shared ethos is currently viewed as limiting choice for women and as a barrier to WCC” [42]. Therefore, WCC applies not only to clinical situations where no interventions are needed but is rather a vision that WCC can still be provided when interventions are necessary as well. This model can include low and high-risk situations by providing a model where needs, wishes and opportunities for decision making can still be applied.

Most of the HCPs who answered the item regarding key indicators of the main perinatal outcome were midwives and obstetricians. Other professionals, such as nurses, paediatricians and anaesthetists, answered that is “not in my activity”. These results show the need to question a possible divide between these two disciplines, with midwives/obstetricians on the one hand, who are concerned with the birth process, and paediatricians/nurses on the other, who are more concerned with the child’s health. From the perspective of anaesthetists, the results suggest that they have a global vision of the mother and child’s health, and probably less knowledge of very specific aspects of childbirth such as episiotomies; however, their underrepresentation in our study suggests that this result should be interpreted with caution. These results show the lack of unification of the WCC concept among HCPs and the need to involve all maternity professionals for cohesion in centred care. The harmonisation of HCPs’ practices around women is one aspect of successful operationalisation in maternity wards.

Our study shows that HCPs have heterogeneous awareness of what defines WCC and its outcomes in perinatal care. Results showed that limited knowledge of each other’s field between the professionals who take care of mothers versus those who take care of newborns, this might be a reason why care centred on the woman, the newborn and the family is insufficiently developed in hospitals. As a result of this research, support and implement the WCC model in a tertiary hospital, would be possible by developing a common philosophy and adapted education with a multidisciplinary approach.

### Strengths and limitations

The strength of this study is that it brought together two scientific methods, quantitative and qualitative. These two approaches which complement each other to analyse and understand the evaluated issue. A challenge in this study, which reinforces the relevance of its results, was to involved all the HCPs working in perinatal care. The fact that the study was carried out in a large hospital facility providing both physiological and complex care allowed the contribution of HCPs working in different types of services to be broadly considered; the respectable rate of participation in this study reinforced the validity of the results, even if a greater participation was anticipated because of the involvement of some medical and nursing team managers in this study.

We acknowledge some limitations. The option to participate in the survey or not could have caused self-selection bias, as the profiles of the HCPs who participated had different characteristics from those of the other HCPs, which limited the generalisability of the results.

Including more doctors in the qualitative part of the study would have allowed a wider exploration of the professionals’ values. The study had to be limited to professionals and did not include pregnant women and their partners which could also have complemented the analysis of the issue. The survey population represents a fragmented view of Switzerland and does not represent other cultural regions of the country, and its analysis is limited to one large hospital.

## Conclusion

Only half of the healthcare professionals, mainly midwives and with a marked difference between medical specialties, had heard of WCC. Most HCPs interviewed agreed with Leap’s definition of WCC. Some HCPs complemented it with their own vision, thus bringing richness and a broader vision of the model. Therefore, a consensual definition integrating views from all HCPs is essential. HCPs need valid scientific knowledge of the benefits of WCC if it were to be implemented. As pointed out, professionals are more oriented to their own perceptions and less to scientific knowledge, as there may be not sufficient high quality evidence or HCPs may not be not aware of existing evidence. This finding applies to all professional categories. The WCC model is still in development. Conceptualised and practical application of a model requires the appropriation by all stakeholders involved in the model development and implementation.

## Supporting information

S1 Table Knowledge and degree of agreement of HCPs with perinatal outcomes of WCC. (PDF)

S2 Table A descriptive analysis by profession of knowledge and degree of agreement of the effect of WCC with perinatal outcomes, positive statements. (PDF)

S3 Table Percentage by professions answering the option ‘not applicable to my activity’. (PDF)

S1 Questionnaire (PDF)
