# Supplementary material for: Exploring health care professionals’ experiences and knowledge of woman-centred care in a university hospital
Source: PLoS One. 2023 Jul 5;18(7):e0286852. doi: 10.1371/journal.pone.0286852 (PMC10321621; doi:10.1371/journal.pone.0286852)
Supplement: S2 Table — (PDF) [file pone.0286852.s002.pdf]

Supplementary table 2: A descriptive analysis by profession of knowledge and degree of agreement of the effect of WCC with perinatal outcomes, positive statements.

Items	All respondent	Midwife	Nurse	Obstetrician	Paediatrician	Anaesthe siologist
	n/N (%)	n/N (%)	n/N (%)	n/N (%)	n/N (%)	n/N (%)
When woman-, newborn- and family-centred care is applied, women adhere more often to the recommended health care pathway	246/252 (97.6)	118/121 (97.5)	79/81 (97.5)	28/29 (96.6)	12/12 (100)	9/9 (100)
Woman-, newborn- and family-centred care promotes spontaneous vaginal birth	102/161 (63.4)	73/106 (68.9)	8/14 (57.1)	13/27 (48.1)	3/5 (60.0)	5/9 (55.5)
When care is centred on the woman, the newborn and the family, women are less likely to have an episiotomy	37/149 (24.8)	25/104 (24.0)	5/10 (50.0)	5/27 (18.5)	1/3 (33.3)	1/5 (20.0)
When care is centred on the woman, the newborn and the family, women are less likely to have an epidural	60/162 (37.0)	49/109 (44.9)	3/14 (21.4)	7/27 (25.9)	1/3 (33.3)	0/9
Woman-, newborn- and family-centred care increases women's satisfaction	250/252 (99.2)	122/123 (99.2)	81/81 (100)	28/29 (96.6)	10/10 (100)	9/9 (100)
Care is woman-, newborn- and family-centred and is accessible to women in vulnerable situations as well as to other women	185/254 (72.8)	84/123 (68.3)	65/83 (78.3)	22/29 (75.8)	8/10 (80.0)	6/9 (66.6)
Woman-, newborn- and family-centred care supports neonatal adaptation	127/212 (59.9)	64/110 (58.2)	42/46 (91.3)	12/28 (42.9)	6/11 (54.5)	3/7 (42.9)
Woman-, newborn- and family-centred care increases the risk of transfers to a neonatal care unit (-)	167/227 (73.6)	87/115 (75.7)	49/65 (75.4)	18/28 (64.3)	7/11 (63.6)	6/8 (75.0)
Practising woman-, newborn- and family-centred care increases HCPs' job satisfaction.	248/266 (93.2)	118/124 (95.2)	86/91 (96.7)	24/29 (82.8)	12/13 (92.3)	8/9 (88.9)
Practising woman-, newborn- and family-centred care	226/264 (85.6)	106/124 (85.5)	78/89 (87.6)	22/29 (75.9)	12/13 (92.3)	8/9 (88.9)

improves HCPs' feeling of professional value						
Woman-, newborn- and family-centred care leads to tensions between different hospital working cultures (-)	88/261 (33.7)	40/122 (32.8)	31/88 (35.2)	9/29 (31.0)	4/13 (30.8)	4/9 (44.4)
Woman-, newborn- and family-centred care increases the risk of professional burnout (-)	185/264 (70.1)	86/123 (69.9)	65/90 (72.2)	16/29 (55.2)	10/13 (76.9)	8/9 (88.9)
Woman-, newborn- and family-centred care reduces hospital costs	95/256 (37.3)	56/123 (53.7)	32/83 (38.6)	7/28 (25.0)	0/13	0/9
