# Supplementary material for: Exploring health care professionals’ experiences and knowledge of woman-centred care in a university hospital
Source: PLoS One. 2023 Jul 5;18(7):e0286852. doi: 10.1371/journal.pone.0286852 (PMC10321621; doi:10.1371/journal.pone.0286852)
Supplement: S3 Table — (PDF) [file pone.0286852.s003.pdf]

Supplementary table 3: Percentage by professions answering the option 'not applicable to my activity' (N=270)

	All Respondent N=270	Midwife n = 124	Nurse n = 95	Obstetrician n = 29	Paediatrician n = 13	Anaesthetist n = 9
	n (%)	n (%)	n (%)	n (%)	n (%)	n (%)
When woman-, newborn- and family-centred care is applied, women adhere more often to the recommended health care pathway	18 (6.7)	3 (2.4)	14 (14.7)	0	1 (7.7)	0
Woman-, newborn- and family-centred care promotes spontaneous vaginal birth	109 (40.4)	18 (14.5)	81 (85.3)	2 (6.9)	8 (61.5)	0
When care is centred on the woman, the newborn and the family, women are less likely to have an episiotomy	121 (44.8)	20 (16.1)	85 (89.5)	2 (6.9)	10 (76.9)	4 (44.4)
When care is centred on the woman, the newborn and the family, women are less likely to have an epidural	108 (40.0)	15 (12.1)	81 (85.3)	2 (6.9)	10 (76.9)	0
Woman-, newborn- and family-centred care increases women's satisfaction	18 (6.7)	1 (0.8)	14 (14.7)	0	3 (23.1)	0
Care is woman-, newborn- and family-centred and is accessible to women in vulnerable situations as well as to other women	16 (5.9)	1 (0.8)	12 (12.6)	0	3 (23.1)	0
Woman-, newborn- and family-centred care supports neonatal adaptation	58 (21.5)	14 (11.3)	39 (41.0)	1 (3.4)	2 (15.4)	2 (22.2)
Woman-, newborn- and family-centred care increases the risk of transfers to a neonatal care unit (-)	43 (15.9)	9 (7.3)	30 (31.6)	1 (3.4)	2 (15.4)	1 (11.1)
Practising woman-, newborn- and family-centred care increases HCPs' job satisfaction.	4 (1.5)	0	4 (4.2)	0	0	0
Practising woman-, newborn- and family-centred care improves HCPs' feeling of professional value	5 (1.9)	0	5 (5.3)	0	0	0

Woman-, newborn- and family-centred care leads to tensions between different hospital working cultures (-)	8 (3.0)	2 (1.6)	6 (6.3)	0	0	0
Woman-, newborn- and family-centred care increases the risk of professional burnout (-)	5 (1.9)	1 (0.8)	4 (4.2)	0	0	0
Woman-, newborn- and family-centred care reduces hospital costs	14 (5.2)	1 (0.8)	11 (11.6)	1 (3.4)	0	1 (11.1)
