# Supplementary material for: Exploring health care professionals’ experiences and knowledge of woman-centred care in a university hospital
Source: PLoS One. 2023 Jul 5;18(7):e0286852. doi: 10.1371/journal.pone.0286852 (PMC10321621; doi:10.1371/journal.pone.0286852)
Supplement: S1 Questionnaire — (PDF) [file pone.0286852.s004.pdf]

ESSIC

EXPÉRIENCE DES SOIGNANTS SUR L'IMPLÉMENTATION DES SOINS CENTRÉS SUR LA FEMME DANS UN HÔPITAL UNIVERSITAIRE

Début du questionnaire

Nous vous remercions par avance pour votre participation. Nous vous invitons à compléter ce questionnaire jusqu'au bout.

Pour chaque question, nous vous demandons de donner votre appréciation et de compléter votre réponse librement. Il n'y a pas de réponse juste ou fausse et seuls votre avis, vos expériences et votre perception ont de l'importance pour l'étude.

Merci pour votre participation

N.B. Pour éviter de recevoir les emails de rappel, vous pouvez nous avertir que vous avez rempli le questionnaire-papier par email à essic@hesav.ch

Partie A: Situation personnelle et professionnelle

A1. Quelle est votre profession ?	☐ Anesthésiste<br>☐ Gynécologue/obstétricien(ne)<br>☐ Infirmier(ère)<br>☐ Infirmier(ère) et sage-femme<br>☐ Pédiatre<br>☐ Sage-femme<br>☐ Autre : ………………………………..
A2. Depuis combien d'années exercez-vous cette profession ?	
A3. Quel est le pays de l'obtention de votre diplôme ?	☐ Suisse<br>☐ Pays de l'Union européenne<br>☐ Hors UE
A4. Avez-vous suivi une formation certifiante ou diplômante après l'obtention de votre diplôme initial ?	☐ Oui<br>☐ Non
A5. Si oui, quel type de formation avez-vous suivi ?	☐ Certificat/CAS<br>☐ Diplôme/DAS<br>☐ Master<br>☐ Doctorat<br>☐ Autre: …………………………….
A6. Depuis combien d'années travaillez-vous aux HUG ?	☐ 0 -1<br>☐ 1-2<br>☐ 2-5<br>☐ 5-10<br>☐ 10 - 20<br>☐ > 20<br>☐ Autre: …………………………………………..
A7. Quel est votre taux d'activité ?	
A8. Quel poste occupez-vous actuellement ?	☐ Cadre (infirmier(ère), sage-femme) / chargé(e) d'encadrement (infiremier(ère), sage-femme)<br>☐ Clinicien(ne)<br>☐ Infirmier(ère)<br>☐ Médecin interne<br>☐ Médecin cadre<br>☐ Sage-femme<br>☐ Autre : ………………………………..

A9. Dans quel service travaillez-vous actuellement ?	☐ Consultations prénatales<br>☐ Prénatal (hospitalisation)<br>☐ Salle d'accouchement<br>☐ Post-partum<br>☐ Urgences gynécologiques et obstétricales<br>☐ Médecine fœtale<br>☐ Accompagnement global<br>☐ Néonatologie<br>☐ Unité de soins intensif de néonatologie (USI)<br>☐ Développement et croissance<br>☐ Autre: ……………………………….
A10. Etes-vous un(e) :	☐ Femme<br>☐ Homme
A11. Quelle est votre année de naissance ?	

Partie B: Connaissances personnelles des soins centrés sur la femme, le nouveau-né et la famille

B1. Avez-vous déjà entendu parler des soins centrés sur la femme, le nouveau-né et la famille ?	☐ Oui<br>☐ Non
B2. Si oui, dans quelle(s) circonstance(s) ?<br><br>(plusieurs réponses possibles)	☐ Lors ma formation de base<br>☐ Lors d'une formation continue<br>☐ Lors de congrès<br>☐ Lors de lecture scientifique<br>☐ Lors de colloque<br>☐ Lors de discussion entre collègues<br>☐ Recherche sur internet<br>☐ Autre : ………………………………….
B3. Quelle serait votre définition des soins centrés sur la femme, le nouveau-né et la famille ?	

Partie C : Définition

La définition des soins centrés sur la femme, le nouveau-né et la famille utilisée pour cette recherche est la suivante :

Les soins centrés sur la femme, le nouveau-né et la famille impliquent que la pratique des soins :

- Se concentre sur les aspirations, les attentes et les besoins individuels de la femme plutôt que sur les besoins de l'institution ou des professionnels
- Reconnaît le besoin des femmes d'avoir le choix, de garder le contrôle et de bénéficier de la continuité d'un ou de plusieurs soignants qu'elle connaît
- Englobe les besoins du bébé, de la famille et des autres personnes importantes pour la femme, telles qu'elle les a elle-même définies
- Assure le suivi de la femme dans les transitions entre les soins de proximité et les contextes de soins aigus
- Répond aux attentes et aux besoins sociaux, émotionnels, physiques, psychologiques, spirituels et culturels de la femme
- Reconnaît les compétences de la femme dans la prise de décision

(Leap, N. (2009). Woman-centred care or women-centred care: does it matter? *British Journal of Midwifery Vol 17* (1) pp 12-16)

C1. Que pensez-vous de cette définition par rapport à ce que vous avez mentionné précédemment ?

Partie D : Composantes des soins centrés sur la femme, le nouveau-né et la famille, et pratique professionnelle

D1. Au cours des 3 derniers mois, à quelle fréquence avez-vous pu mettre en pratique les différentes composantes des soins centrés sur la femme, le nouveau-né et la famille issues de la définition donnée précédemment ?					
	Jamais	Parfois	Souvent	Toujours	Ne s'applique pas à mon activité
Les soins se concentrent sur **les besoins (nécessité primaire)** de la femme et non sur ceux de l'institution ou des professionnels					
La femme a **le choix** dans les soins pour elle et son bébé					
Les soins se concentrent sur **les attentes (souhait)** de la femme et non sur celles de l'institution ou des professionnels					
Les soins répondent aux **attentes et besoins socio-culturels** de la femme					
La femme bénéficie **de la continuité** de la prise en charge avec un soignant qu'elle connaît					
Les soins se concentrent sur **les aspirations (idéal)** de la femme et non sur celles de l'institution ou des professionnels					
La femme est **partie prenante** des soins pour elle et son bébé					
Les soins englobent les besoins **du bébé**					
Les soins englobent les besoins **de la famille**					
**Le suivi de la femme et de son bébé** est assuré entre les soins extra et intra hospitaliers					
Les soins répondent aux **attentes et besoins spirituels** de la femme					
Les soins englobent les besoins **des personnes qui-sont proches de la femme**					
Les compétences de la femme dans la **prise de décision** sont reconnues durant la grossesse					
Les soins répondent aux **attentes et besoins psycho-émotionnels** de la femme					
Les compétences de la femme dans la **prise de décision** sont reconnues durant le travail et l'accouchement					
Les soins répondent aux **attentes et besoins physiques** de la femme					
Les compétences de la femme dans la **prise de décision** sont reconnues durant le post-partum					

Partie E : Composantes des soins centrés sur la femme, le nouveau-né et la famille, et perceptions

E1. Au sujet des soins centrés sur la femme, le nouveau-né et la famille, à quel point êtes-vous d'accord avec les affirmations suivantes ?						
	Pas du tout d'accord	Pas d'accord	Ni d'accord ni pas d'accord	Plutôt d'accord	Tout à fait d'accord	Ne s'applique pas à ma pratique
Lorsque les soins sont centrés sur la femme, le nouveau-né et la famille, la femme **adhère plus facilement** aux recommandations pour sa santé et celle de son enfant						
Les soins centrés sur la femme, le nouveau-né et la famille **favorisent l'accouchement spontané par voie basse**						
Lorsque les soins sont centrés sur la femme, le nouveau-né et la famille, la femme a **moins de risque** d'avoir une épisiotomie						
Lorsque les soins sont centrés sur la femme, le nouveau-né et la famille, la femme a **moins souvent recours à la péridurale**						
Les soins-centrés sur la femme, le nouveau-né et la famille **augmentent la satisfaction** des femmes						
Les soins sont centrés sur la femme, le nouveau-né et la famille sont accessibles autant aux femmes en **situation de vulnérabilité qu'aux autres femmes**						
Les soins centrés sur la femme, le nouveau-né et la famille **améliorent l'adaptation de l'enfant** à la vie extra utérine						
Les soins centrés sur la femme, le nouveau-né et la famille **augmentent le risque pour le nouveau-né** d'être transféré en néonatologie						

E1 suite : Au sujet des soins centrés sur la femme, le nouveau-né et la famille, à quel point êtes-vous d'accord avec les affirmations suivantes ?						
Pratiquer des soins centrés sur la femme, le nouveau-né et la famille **augmente la satisfaction professionnelle** des soignants						
Pratiquer des soins centrés sur la femme, le nouveau-né et la famille **améliore le sentiment de reconnaissance professionnelle** des soignants						
Les soins centrés sur la femme, le nouveau-né et la famille entraînent des **tensions entre différentes cultures de travail** hospitalières						
Les soins centrés sur la femme, le nouveau-né et la famille **augmentent le risque de burnout** au travail						
Les soins centrés sur la femme, le nouveau-né et la famille **diminuent les coûts** hospitaliers						

E2. Voudriez-vous ajouter quelque chose à la liste précédente ?

Partie F : Barrières à l'implémentation des soins centrés sur la femme, le nouveau-né et la famille

F1. Dans la liste ci-dessous, indiquer à point chaque élément est selon vous une barrière à la mise en pratique des soins centrés sur la femme, le nouveau-né et la famille, dans votre pratique

	Pas une barrière 0	1	2	3	Barrière insurmontable 4
**Manque de documents de référence** sur les soins centrés sur la femme, le nouveau-né et la famille					
Tradition de pratique **biomédicale** (centrée sur le risque et la pathologie)					
**Manque de flexibilité** dans l'institution					
Enjeux de **pouvoir dans les relations** professionnelles					
Manque d'**intimité** des lieux de soins					
Ecoute active entravée par l'utilisation de l'**ordinateur**					
**Dossier de soins** gardé par l'hôpital et non par la femme					
**Manque de temps pour se former** aux soins centrés sur la femme, le nouveau-né et la famille					
**Trop de travail** pour mettre en place des soins centrés sur la femme, le nouveau-né et la famille					
Plus de **moyens (logistiques, personnel…) nécessaires** pour les soins centrés sur la femme, le nouveau-né et la famille que pour les soins habituels					
**Tournus et renouvellement fréquent** des soignants					
**Scepticisme** des soignants sur la valeur des soins centrés sur la femme, le nouveau-né et la famille					
**Manque de compréhension** de ce que sont des soins centrés sur la femme, le nouveau-né et la famille					
Barrière de la **langue**					
Courte durée du **passage des femmes** dans l'institution					
Situations d'**urgence**					
Manque de **connaissance** des soins centrés sur la femme, le nouveau-né et la famille par les femmes et leurs proches					

F2. Existe-t-il d'autres barrières à l'implémentation des soins centrés sur la femme, le nouveau-né et la famille à la maternité des HUG ?

Partie G : Les facilitateurs à l'implémentation des soins centrés sur la femme, le nouveau-né et la famille

G1. Dans la liste ci-dessous, indiquer à point chaque élément est selon vous un facilitateur à la mise en pratique des soins centrés sur la femme, le nouveau-né et la famille, dans votre pratique					
	Pas un facilitateur 0	1	2	3	Fort facilitateur 4
Compréhension personnelle de la **valeur des soins** centrés sur la femme, le nouveau-né et la famille					
Compréhension personnelle des **tâches spécifiques** aux soins centrés sur la femme, le nouveau-né et la famille					
**Compréhension commune** à tous les soignants des buts des soins centrés sur la femme, le nouveau-né et la famille					
**Perception des différences** entre les soins centrés sur la femme, le nouveau-né et la famille et les autres méthodes de travail					
Présence de **professionnels reconnus comme experts** qui mettent en avant les soins centrés sur la femme, le nouveau-né et la famille					
**Engagement sur la durée** des soignants dans les soins centrés sur la femme, le nouveau-né et la famille					
Ouverture à travailler avec d'autres soignants sur des **nouveaux moyens** pour utiliser les soins centrés sur la femme, le nouveau-né et la famille					
Sentiment de **légitimité** pour participer aux soins centrés sur la femme, le nouveau-né et la famille					
Existence de **formations** dans les soins centrés sur la femme, le nouveau-né et la famille pour les soignants					
Attribution du travail aux soignants qui ont les **compétences** pour effectuer des soins centrés sur la femme, le nouveau-né et la famille					
Possibilité d'**intégrer facilement** les soins centrés sur la femme, le nouveau-né et la famille dans mon travail actuel					
**Confiance en la capacité des autres** à utiliser les soins centrés sur la femme, le nouveau-né et la famille					
**Infrastructure** qui permet de dispenser des soins centrés sur la femme, le nouveau-né et la famille					
**Politiques institutionnelles** qui soutiennent la mise en pratique des soins centrés sur la femme, le nouveau-né et la famille					
Possibles **effets positifs** des soins centrés sur la femme, le nouveau-né et la famille sur mon travail					

G1. Suite : Dans la liste ci-dessous, indiquer à point chaque élément est selon vous un facilitateur à la mise en pratique des soins centrés sur la femme, le nouveau-né et la famille, dans votre pratique					
	Pas un facilitateur 0	1	2	3	Fort facilitateur 4
Reconnaissance de la **plus-value** des soins centrés sur la femme, le nouveau-né et la famille par les soignants					
**Evaluation continue de la qualité** en vue d'améliorer les soins centrés sur la femme, le nouveau-né et la famille					
**Accessibilité des recherches** menées sur les soins centrés sur la femme, le nouveau-né et la famille					
Connaissance des soins centrés sur la femme, le nouveau-né et la famille **par les femmes et leurs proches**					
Implication de **toutes les personnes concernées** dans la mise en place des soins centrés sur la femme, le nouveau-né et la famille (soignants, femmes, proches, décideurs)					
Considérer les femmes comme **capables de s'impliquer** dans les soins centrés sur la femme, le nouveau-né et la famille					

G2. Existe-t-il d'autre facilitateurs à l'implémentation des soins centrés sur la femme, le nouveau-né et la famille à la maternité des HUG ?

Merci d'avoir complété ce questionnaire jusqu'au bout !

Pensez à le glisser dans l’enveloppe ci-jointe et à l’envoyer par courrier interne à :

Etude : ESSIC
Expérience des soignants sur l’intégration des soins centrés sur la femme, le nouveau-né et la famille
Casier de Lucia Floris
c/o MATERNITE HUG

Ou par courrier postal à :

Lucia Floris
HESAV
Avenue de Beaumont 21
1011 Lausanne

Si vous désirez participer à un entretien, vous pouvez laisser votre adresse email ci-dessous ou nous contacter par email à essic@hesav.ch

Adresse email : ………………………………………………………………………………………………………

Nous vous remercions de votre précieuse participation et vous encourageons à parler de cette étude autour de vous afin que vos collègues pensent également à remplir ce questionnaire !
